# Nitrogen-Deficiency Stress Induces Protein Expression Differentially in Low-N Tolerant and Low-N Sensitive Maize Genotypes

**DOI:** 10.3389/fpls.2016.00298

**Published:** 2016-03-21

**Authors:** Muslima Nazir, Renu Pandey, Tariq O. Siddiqi, Mohamed M. Ibrahim, Mohammad I. Qureshi, Gerard Abraham, Krishnapriya Vengavasi, Altaf Ahmad

**Affiliations:** ^1^Department of Botany, Faculty of Science, Jamia HamdardNew Delhi, India; ^2^Division of Plant Physiology, Indian Council of Agricultural Research-Indian Agricultural Research InstituteNew Delhi, India; ^3^Department of Botany and Microbiology, Science College, King Saud UniversityRiyadh, Saudi Arabia; ^4^Department of Botany and Microbiology, Faculty of Science, Alexandria UniversityAlexandria, Egypt; ^5^Department of Biotechnology, Faculty of Natural Sciences, Jamia Millia IslamiaNew Delhi, India; ^6^Centre for Conservation and Utilization of Blue Green Algae, Indian Council of Agricultural Research-Indian Agricultural Research InstituteNew Delhi, India; ^7^Department of Botany, Faculty of Life Sciences, Aligarh Muslim UniversityAligarh, India

**Keywords:** nitrogen stress, *Zea mays*, proteome, 2DE, nitrogen use efficiency

## Abstract

Nitrogen (N) is essential for proper plant growth and its application has proven to be critical for agricultural produce. However, for unavoidable economic and environmental problems associated with excessive use of N-fertilizers, it is an urgent demand to manage application of fertilizers. Improving the N-use efficiency (NUE) of crop plants to sustain productivity even at low N levels is the possible solution. In the present investigation, contrasting low-N sensitive (HM-4) and low-N tolerant (PEHM-2) genotypes were identified and used for comparative proteome-profiling of leaves under optimum and low N as well as restoration of low N on 3rd (NR3) and 5th (NR5) days after re-supplying N. The analysis of differential expression pattern of proteins was performed by 2-D gel electrophoresis. Significant variations in the expression of proteins were observed under low N, which were genotype specific. In the leaf proteome, 25 spots were influenced by N treatment and four spots were different between the two genotypes. Most of the proteins that were differentially accumulated in response to N level and were involved in photosynthesis and metabolism, affirming the relationship between N and carbon metabolism. In addition to this, greater intensity of some defense proteins in the low N tolerant genotype was found that may have a possible role in imparting it tolerance under N starvation conditions. The new insights generated on maize proteome in response to N-starvation and restoration would be useful toward improvement of NUE in maize.

## Introduction

Present environmental scenario of the globe and the concurrent need for adequate food productivity makes it essential to consider an approach that sustains the environment and the food economy at the same time. Fertilizer application has been one of the key factors in improving crop productivity. However, excessive use of fertilizer application is causing severe environmental problems. Therefore, need of the hour is to reduce the use of fertilizers without any penalty on crop productivity (Hakeem et al., 2013). Being the constituting element of amino acids, proteins (enzymes), nucleic acids, chlorophyll and various plant hormones, nitrogen (N) is classified as a crucial plant macronutrient. Nitrogen availability affects processes like growth, development, architecture, flowering, senescence, photosynthesis and allocation of photosynthates in a plant (Prinsi et al., 2009). For agricultural crop production, N is applied in the form of various nitrogenous fertilizers (Prasad, 1998). World-wide nitrogenous fertilizer consumption is 113.06 million tons (FAO, 2011). In India alone the consumption of fertilizer is 14 million tons a year (FAI, 2014) and is still on a rise, growing 5% annually (Pathak et al., 2008). However, it is worth concern that the agricultural crop plants specially wheat, rice and maize utilize only 30–40% of the applied N. Rest of the 60–70% of applied N remains unutilized at the agricultural field, causing severe environmental and health hazards (Hakeem et al., 2011). Every year 100 teragram of nitrogen is released into the environment in the form of nitrous oxide from the agricultural fields. The nitrous oxide has 300 times more global warming effect than carbon dioxide (EPA, 2010).

Improving the N-use efficiency (NUE) in crop plants could help us achieve the target of increasing crop productivity and the development of an environment friendly cropping system. From agronomic methods to transgenic efforts, many approaches were used to deal with this issue. Split application of N, slow release of fertilizers and use of nitrification inhibitors were some of the agronomic techniques used (Hirel and Lea, 2001; Pathak et al., 2008). Many researchers have attempted to expound the regulatory control mechanisms involved during transition of nitrate from source to sink organs. These approaches, however, involve the study of whole plant physiology and help in identifying the role of a single or limited number of enzymes or regulatory elements and do not provide a detailed account for the variation of the complex trait NUE (Hirel et al., 2001; Pathak et al., 2008). Improvement of grain yield through conventional breeding procedures by selecting the most appropriate traits has also been performed (Daubresse et al., 2010). Although this approach was successful in enhancing yield, it could not provide an insight into the genetic basis of these improvements in relation to NUE. Overexpression of critical candidate genes to test their effects on biomass and plant N status was tried by many workers (Crété et al., 1991; Takahashi et al., 2001; Andrews et al., 2004). An increase in nitrate influx was observed by the overexpression of HATS like NRT2.1, but there was no significant change in the phenotypic NUE or nitrate utilization (Fraisier et al., 2000). Similarly, the over-expression of nitrate reductase (NR) and nitrite reductase (NiR) in *Arabidopsis*, potato or tobacco resulted in reduction of nitrate levels in plant tissues but no significant improvement in terms of biomass or seed yield was found (Pathak et al., 2008). Successful effects on plant biomass and grain yield were, however, obtained by the overexpression of cytoplasmic glutamine synthetase 1 (GS1) (Hoshida et al., 2000) and glutamine synthetase 2 (GS2) (Habash et al., 2001) using different promoter combinations. The overexpression of DOF1 (DNA binding with one finger) transcription factor resulted in enhancement of both plant growth and N content under low N conditions (Yanagisawa, 2004). Furthermore, it was found that the expression of EcDof1 (finger millet binding with one finger) to overlap the expression of NR, GS and glutamate synthase (GOGAT) indicating that Dof1 probably regulates the expression of these genes under different conditions by sensing the N fluctuations around the root zone (Gupta et al., 2009). Under low N the gene expression profile of Dof 1 coincided with the enzyme activities of GS, GOGAT (Kumar et al., 2009). These studies suggest that directed changes in just one component of N network may not be enough to effect significant changes in the overall NUE because a large number of genes and interacting pathways are associated with the actual NUE of a plant (Moose and Below, 2009). The utilization of different transcription factors like the cis-elements and trans-acting factors might be therefore considered a good alternative for improving NUE.

It is being speculated that some of these factors act as master switches in the regulation of genes involved in carbon and N metabolism. Once, such master regulators are identified and validated, the complexity of NUE trait will be simplified. For identification of such candidate genes, proteomic approach can be an effective tool. Although, the same complement of genome is present in all the tissues and organs of a plant, it is the expression of genes and accumulation of proteins thereof that varies widely. Proteomics can therefore, provide us with a biological snapshot of a tissue, organ or organelle at a particular point of time. Thus, by exploiting the property of differential expression of proteins it is possible to find out the genes and regulatory switches underlying the NUE mechanism of a crop. Moreover, 2D-gel electrophoresis (2DE) eliminates the limitations encountered in DNA/RNA analysis because of post-translational modifications. A grain of immense agricultural importance, globally maize (*Zea mays* L.) is grown in about 100 million hectares of land, 160 million hectares of maize were harvested in 2010 (FAO, 2010). Survey shows that in developing countries, maize production is projected to grow at 2.2% per annum leading to a corresponding increase in maize cultivation area by 36% (FAO, 2011). In USA, 9 out of the 11 highest N fertilizer using states is the maize producing states (EWG, 1996). In India also, maize is grown on a large scale and the nitrogenous fertilizer applied to corn accounts for more than 30% of the total nitrogenous fertilizer used (FAI, 2014). Given this, the proteomics approach could be helpful in the identification of candidate gene(s) involved in low-N tolerance in maize. Such gene(s) could be used for the development of N efficient crop plants i.e., the variety that can grow well under low N availability.

## Materials and methods

### Plant growing condition and identification of low-N sensitive and low-N tolerant genotypes

Thirty-two maize (*Zea mays* L.) genotypes (Supplementary Table 1) comprising of inbreds, hybrids and composites were procured from the ICAR-Indian Institute of Maize Research and Division of Genetics, ICAR-Indian Agricultural Research Institute, New Delhi. After surface sterilization with 0.1% HgCl_2_, seeds were soaked in 0.1 mM CaCl_2_ solution in dark for 2 days with continous circulation of air. The seeds were then wrapped in paper towels and kept at 30°C CaCl_2_ solution for 5 days. Once the coleoptiles emerged, plants were transferred to plastic containers with thermocol sheet (2 inch thickness). Each container was filled with 10 L of Standardized Hoagland's solution (half strength; Hoagland and Arnon, 1950) for a period of 3 days initially and was replaced by full-strength solution afterwards. Fresh solution was added every 3 days until appearance of the deficiency symptoms. The nutrient (Hoagland's) solution preparation was standardized beforehand and was applied as; low N (50 μM) and optimum N (4.5 mM). As such, the plants had no nutrient-deficiency symptoms other than those due to N, when grown in hydroponics. The composition of nutrient solution was: NH_4_NO_3_ (concentration as per treatment), H_3_PO_4_ (0.5 mM), CaCl_2_ (2.25 mM), MgSO_4_ (0.75 mM), KCl (2.4 mM), NaCl (1 mM) H_3_BO_3_ (0.05μM), MnCl_2_ (0.01μM), ZnSO_4_ (0.002 μM), CuSO_4_ (0.0015 μM), NH_4_Mo_7_O_24_(0.000075 μM), and Fe-EDTA (0.074 μM). The nutrient solution was maintained at pH 5.6. The solution was constantly circulated with fresh air using aquarium pumps all through the experiment. The plants were grown in a glasshouse at National Phytotron Facility, New Delhi, with optimum temperature (30°C/20°C D/N), relative humidity 70% and light (natural) conditions. Specific deficiency symptoms of N stress were visible after 15 days of transfer to nutrient solution.

Screening of the maize genotypes for N starvation tolerance was done on the basis of various physiological and biochemical parameters that were observed for the 15-day-old plants using various accepted methods, *viz*. shoot and root biomass, leaf area, root-to-shoot ratio, root length, photosynthesis rate, chlorophyll content, tissue N concentration, total N uptake and utilization efficiency (NUE). Assays were also performed for the main N assimilation enzymes like nitrate reductase (NR) and glutamine synthetase (GS) in order to assess NUE. The experiment wascarried out in an absolutely randomized pattern with two-factor factorial, treatments as main plot and genotypes as split-plot. Each experiment was replicated thrice. The data obtained from screening experiment were subjected to statistical analysis to select contrasting genotypes. Two-way analysis of variance (ANOVA) was done using the SAS programme. Statistical significance was determined at 1% probability level. Means were compared by the critical difference (CD at *p* = 0.05) following a significant *F*-test. Principal component analysis (PCA) and hierarchical clustering (HC) was performed in R using the functions “*princomp”* and “*hclust*” respectively. The contribution of individual trait to total variability was determined by PCA. Genotypes were classified for N starvation tolerance by *Ward's* method of HC of the squared Euclidean distance matrix of 15 traits grown under low (50 μM) nitrogen.

### Leaf sampling under low-N and N restoration conditions

From the screening experiment, two contrasting genotypes PEHM-2 (V1, low-N tolerant) and HM-4 (V2, low-N sensitive) were identified. These genotypes were again grown under similar conditions as mentioned above with low (50 μM) and optimum (4.5 mM) N concentration. A total of three sets of treated plants were maintained for both N concentrations. On 15th day after transplanting, leaf samples were collected from one set of plants grown with optimum N and low N. The second fully expanded leaf was sampled uniformly for each treatment. In other two sets of low N grown plants, N was added at the rate of 4.5 mM for restoration of N stress on the 15th day. Leaf samples were collected on third (NR3) and fifth (NR5) day of N replete condition. Similarly, leaf tissues were also collected from optimum N plants on the corresponding days to compare the proteome profiles. Collected leaf samples were promptly dipped in liquid nitrogen for freezing and stored at −80°C for further use. Sampling was performed in two biological replicates from three independent experiments.

### Protein isolation and preparation

Protein extraction from leaf samples was performed by the phenol method of Isaacson et al. (2006). Leaf material (2 g) was ground to fine powder using liquid nitrogen and suspended in 10 ml of extraction buffer containing 4-(2-hydroxyethyl)-1-piperazineethanesulfonic acid (HEPES), β-mercaptoethanol, sucrose and phenylmethanesulfonylfluoride (PMSF). 15 ml of Tris-HCl saturated phenol was added and the solution was mixed in a rocker for 30 min and subjected to centrifugation at 5000 rpm for 10 min at 4°C. The upper phenolic phase was carefully recovered in a separate tube and incubated overnight at −20°C for precipitation after adding 15 ml of ice-cold 0.1 M ammonium acetate-methanol solution. The proteins were pelleted by centrifuging at 10,000 rpm for 15 min at 4°C. Protein pellet was washed twice with ice-cold methanol followed by washing with chilled acetone. The resulting pellet was centrifuged at 5000 rpm after each washing, dried and solubilised in buffer containing 2 M thiourea, 7 M urea, 4% (w/v) CHAPS, 50 mM DTT. The protein was quantified using Bradford's reagent (Bio-Rad, USA).

### Two-dimensional gel electrophoresis

Two-dimensional electrophoresis of proteins was performed in accordance with the method of O'Farrell (1975). An immobiline dry strip gel (17 cm, pH 4-7; Bio-Rad, USA) was rehydrated at 20°C for 14 h in 200 μl of sample containing 500 μg of protein. Isoelectric focussing (first-dimensional separation) was carried out in a PROTEAN IEF apparatus (BioRad, USA). The voltages applied were 250 V for 1 h, 500 V for 1 h, 1000 V for 2 h, 2000 V for 2 h, linear increase of 8000 V for 18 h and 500 V for 1 h. After the completion of IEF, strips were subjected to reduction for 15 min by the reduction buffer and then to alkylation buffer for 15 min as mentioned in Bagheri et al. (2015). The SDS-PAGE was carried out in a Dodeca cell (PROTEAN plus, Bio-Rad, USA) for the second-dimensional separation of focussed proteins using 12% SDS at a constant voltage of 100 V. The gels were stained by colloidal Coommassie brilliant blue dye followed by destaining.

### Gel analysis

The gels obtained were scanned by densitometer (GS-800 Calibrated Densitometer, Bio-Rad, USA) and the images thus generated were then analyzed with the help of PD Quest software (Advanced version 8.0 Bio-Rad, USA) for the detection of spots, subtraction of background and quantificationof the intensites. The gel having the maximumt spot number was marked as the reference gel. The spot comparison and analysis for all other gels were corresponded with the reference gel and the spots were matched across all the gels. Each spot value was normalized in terms of percentage of the total volume of all gel spots. This was done to balance the unevenness due to quantitative disparity in spot intensities. The spots with 2-fold change in their volumes during the treatment or a significant variation between the control and other treatments as per the results of paired Student's *t*-test (*p* ≤ 0.05) were spotted as the treatment-responsive proteins.

### In-gel digestion and protein identification

The protein spots were excised from gels, washed and dehydrated with acetonitrile and ammonium bicarbonate and then reduced by 15 mM DTT at 60°C for 1 h. The gel slices were alkylated by 100 mM isoamyl alcohol in dark for 15 min, rehydrated with ammonium bicarbonate and then dried-up in a speed vac for 15 min. The dried gel slices were rehydrated with 15 μl of trypsin (Sequencing grade, Promega, USA) at 37°C for overnight. The supernatant was taken and 20% acetonitrile and 1% formic acid were added to the remaining gel slice for further extraction. The final supernatant was dried in speed vac until the volume was reduced to 25–50 μl. This final volume was analyzed on AB Sciex (Applied Biosciences, USA) MALDI-TOF MS. Peptide tolerance of 150 ppm, fragment mass tolerance of ±0.4 Da, and peptide charge of 1+ were selected during protein database searches performed on a local Mascot (Matrix Science, London, UK) server. On the basis of MASCOT probability analysis (*p* < 0.05) significant hits were accepted. Peptides were searched with reference to NCBI database, taxonomy of green plants, trypsin of digestion enzyme, one missed cleavage site, partial modification of cysteine carboamidomethylated and methionine oxidized.

### Statistical analyses

Three biological replicates for both treatments and control were taken during whole of the experimental course for the statistical analyses. A two-tailed Students *t*-test having 95% significance was performed on the normalized value of protein spots using the SPSS software.

## Results

### Identification of low-N sensitive and low-N tolerant genotypes by PCA and cluster analysis

**Figure 1:**
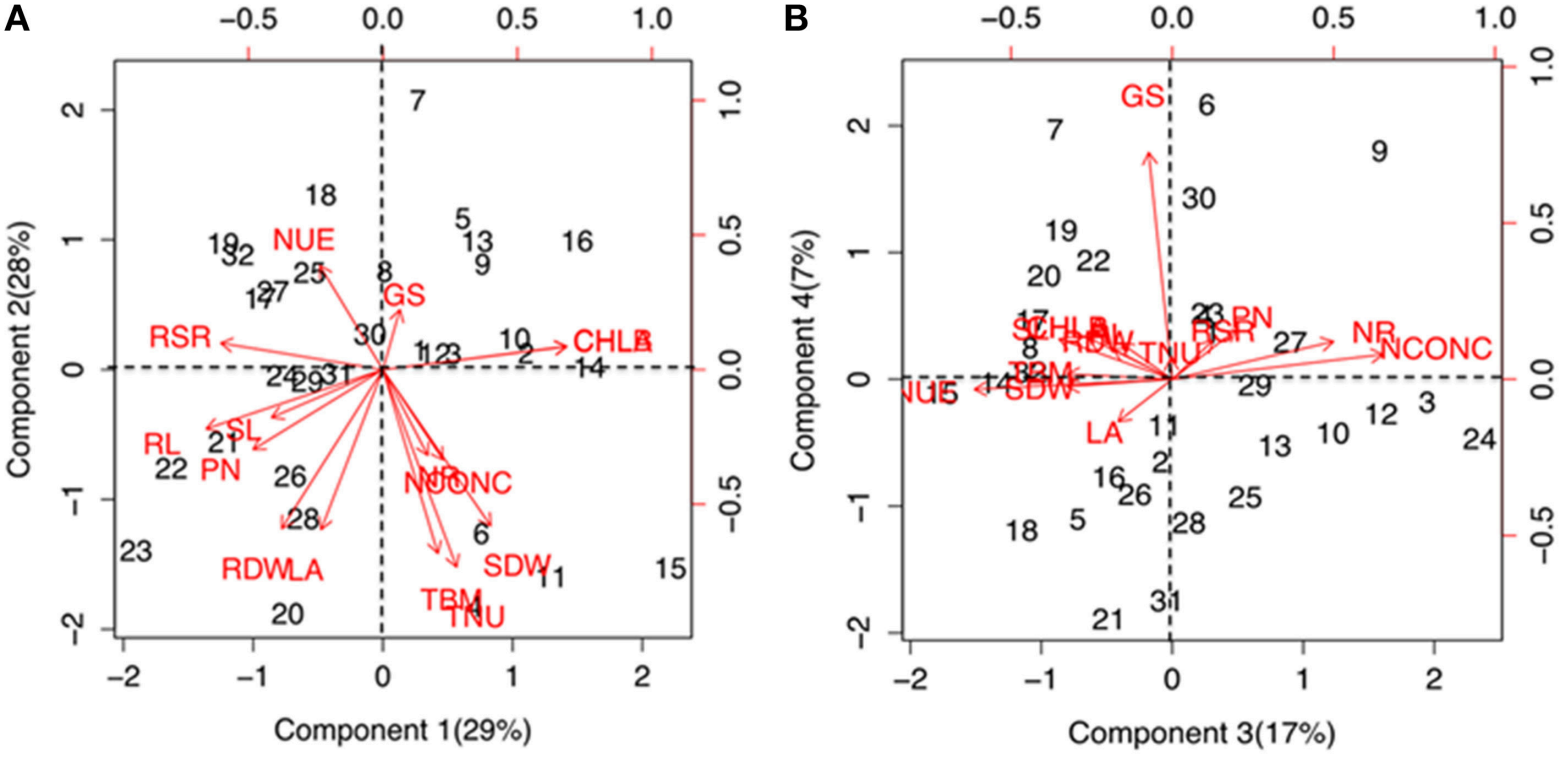
**Biplot showing principal component scores (A) PC1, PC2 and (B) PC3, PC4 of traits governing nitrogen starvation tolerance in maize genotypes grown under low (50 μM) N**. The factor loading values for traits are indicated by red arrows radiating from the center showing the direction (angle) and magnitude (length). Data labels indicate 32 maize genotypes as mentioned in Supplementary Table 1. Legend for traits: SL, shoot length; RL, root length; SDW, shoot dry weight; RDW, root dry weight; TBM, total biomass; RSR, root-to-shoot ratio; LA, leaf area; CHL A, chlorophyll a; CHL B, chlorophyll b; PN, photosynthesis; NCONC, N concentration; TNU, total N uptake; NUE, N use efficiency; NR, nitrate reductase; GS, glutamine synthetase.

Significant (*P* ≤ 1%) differences were observed for various physiological and biochemical traits among the maize genotypes grown under low-N and sufficient N concentrations (Supplementary Table 2). Growth performance at sufficient and low N levels were analyzed by principal component and cluster analysis by taking into consideration 15 trait variables as mentioned above. The principal component vectors, PC1 and PC2 accounted for 57% of the total variation under low-N (Figure 1A). Among the various traits in PC1, the maximum variation was explained by chlorophyll (17.03%), root length (15.7%), root-to-shoot ratio (13.3%) and photosynthesis (8.5%). In PC2, maximum variability was observed for total N uptake (20.3%), followed by total biomass (17.6%), total leaf area (13.3%), root (13.2%) and shoot (12.7%) dry weight. 24% of the total variation under low-N was attributed to PC3 and PC4 (Figure 1B). In PC3, the maximum variation was explained by tissue N concentration (25.5%) followed by N use efficiency (22.5%) and NR activity (14.9%) while in PC4, GS activity alone (76.0%) governed the maximum variability. Figures 1A,B also shows the positive and negative scores of all principal component vectors. The traits that added up to the maximum variability were present in +PC1 and −PC2, and were classified as tolerant. From this quadrant, PEHM-2 was identified as N starvation tolerant genotype (Supplementary Figure 1). In +PC1 and +PC2, chlorophyll and GS activity were present which did not contribute significantly toward variability and was classified as N starvation sensitive. HM-4 was identified as N starvation sensitive genotype.

**Figure 2:**
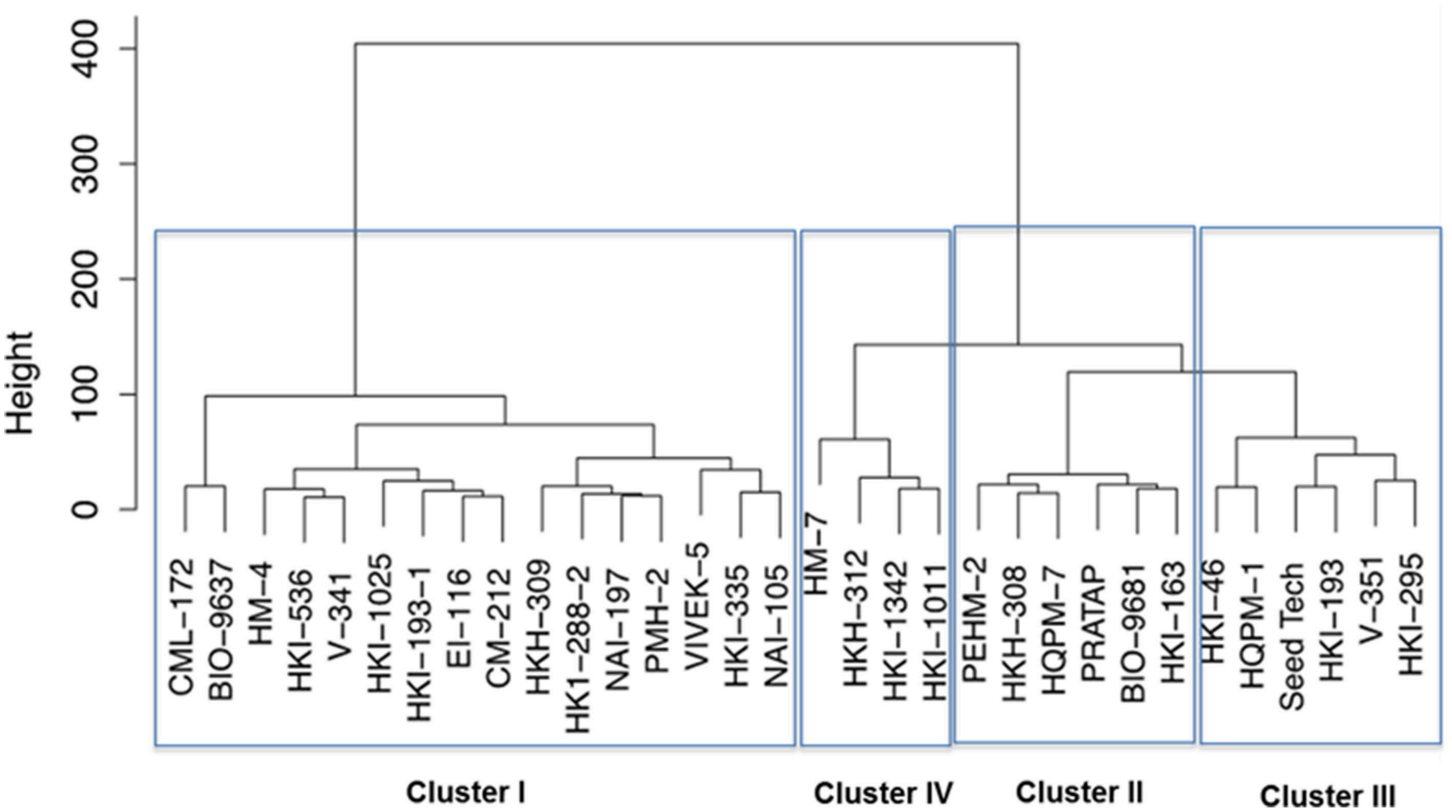
**Dendrogram of maize genotypes classified by *Ward's* method of hierarchical clustering of the squared Euclidean distance matrix of 15 traits grown under low (50 μM) nitrogen**.

Further, the genotypes were grouped into four clusters by the use of the Euclidean distances between genotypes significantly varying in growth parameters at N starvation condition (Figure 2). Out of four clusters, lowest cluster means in almost all the important traits was identified for 16 genotypes belonging to cluster I by PCA. Six genotypes of cluster II had high mean values for shoot and total plant biomass, chlorophyll, NR activity, tissue N concentration and total N uptake. Cluster III and IV comprised of six and four genotypes, respectively. Cluster III genotypes possessed maximum mean values for root length, root-to-shoot ratio, NUE and photosynthesis while those in cluster IV had highest mean values for root biomass and leaf area. The cluster II genotypes were found to be tolerant and genotypes belonging to cluster I were sensitive to N starvation. Thus, PEHM-2 genotype from cluster II and HM-4 genotype from cluster I was selected for proteomic studies under low-N and restoration conditions.

### Leaf proteome analysis of contrasting maize genotypes

**Figure 3:**
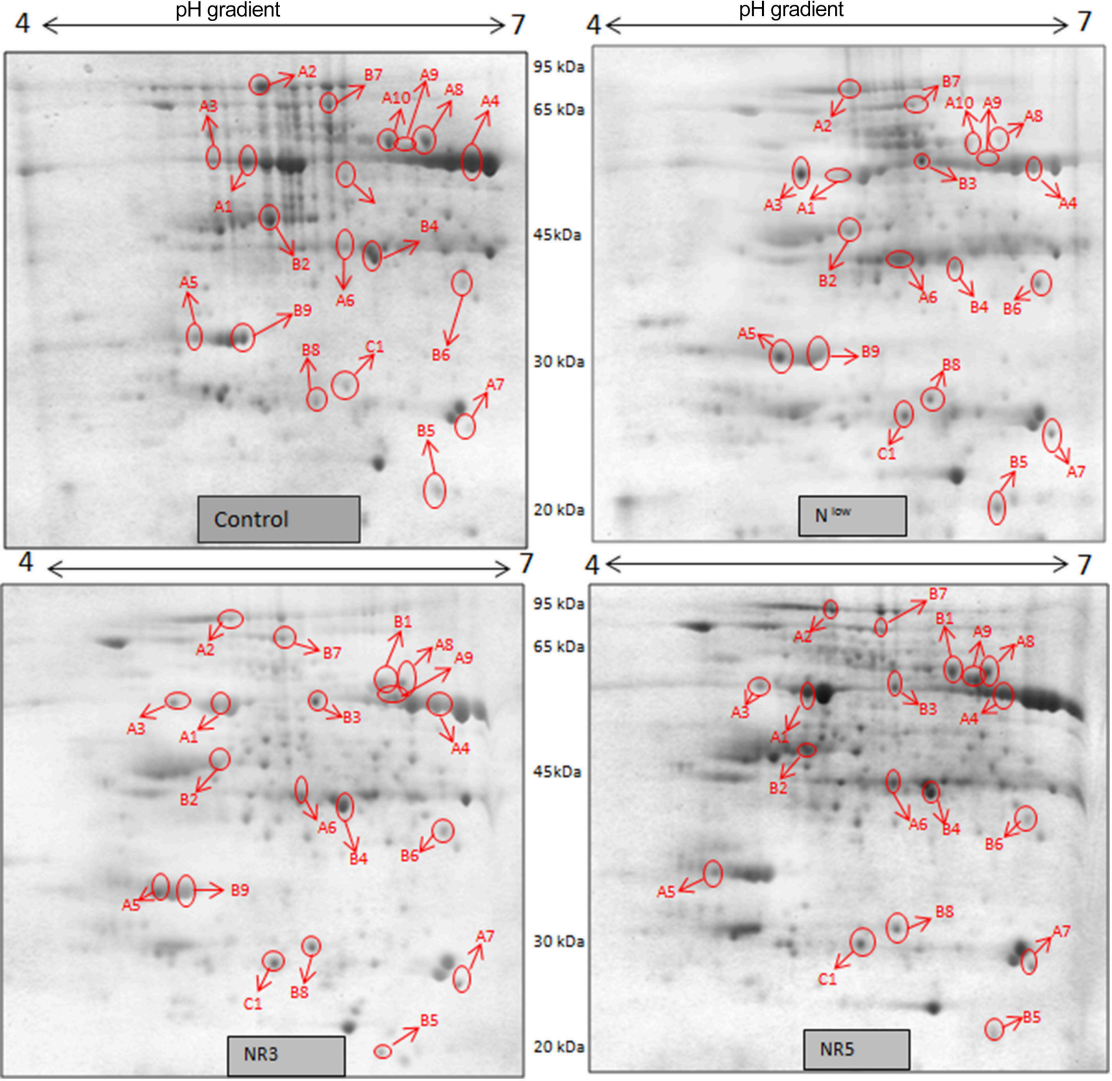
**2DE maps representing differential protein intensities between different N treatments of PEHM-2**.

**Figure 4:**
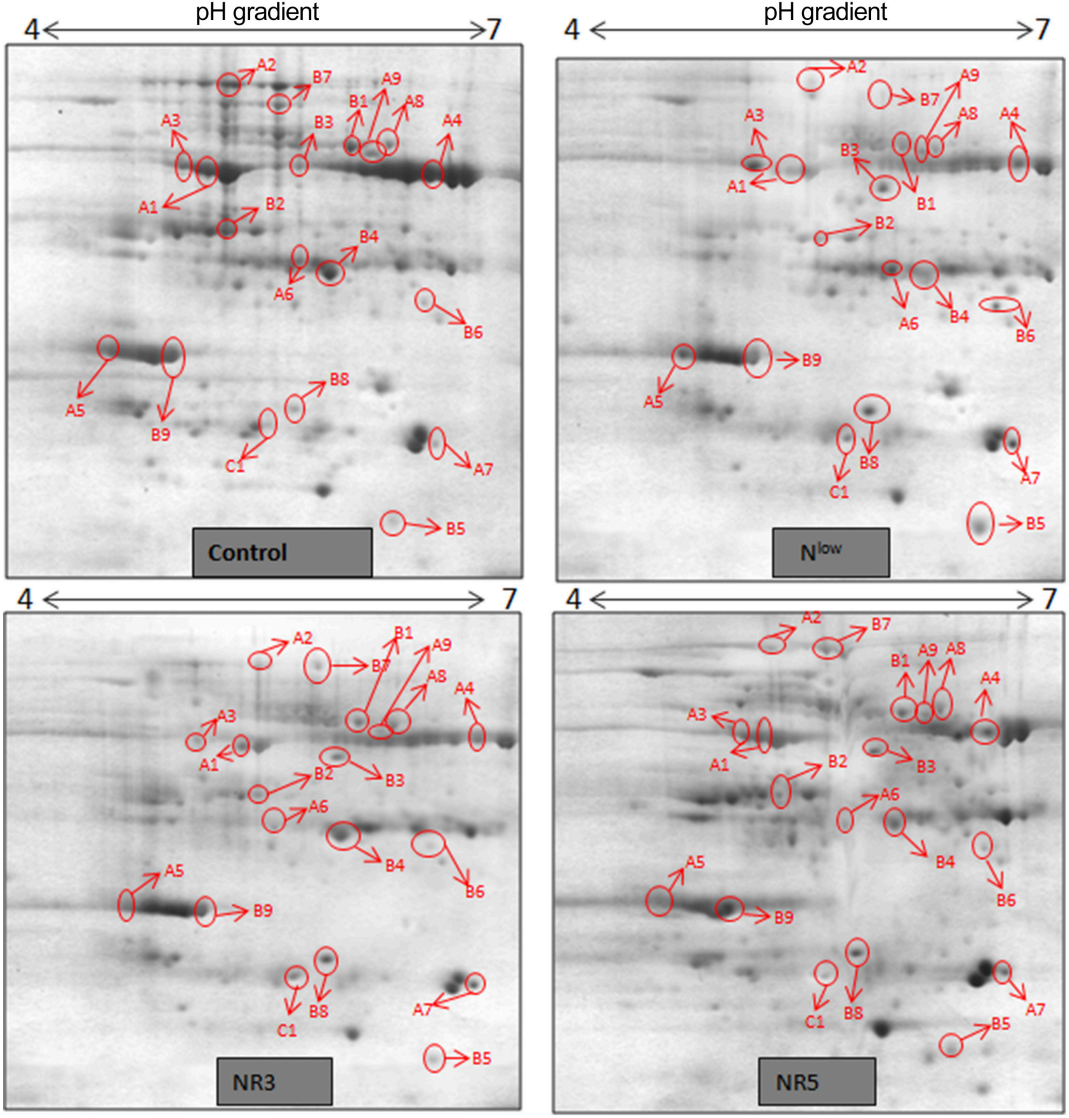
**2DE maps representing differential protein intensities between different N treatments of HM-4**.

Gel based proteomics was adopted in this study to resolve the differentially expressed proteins of low-N tolerant and low-N sensitive maize genotypes (Supplementary Figure 2) under various levels of N to identify the candidate proteins responsible for regulation of N uptake. Protein profiles of leaf samples with optimum-N, low-N and low-N followed by N-restoration were compared for each genotype separately. Further, an inter-genotype comparison was done by analyzing the control profiles of the two genotypes. Isoelectric points of the analyzed gels ranged from 4 to 7. In total, 324 and 316 spots were detected in the 2D profiles of control samples of PEHM-2 and HM-4, respectively (Table 1). A synthetic gel pattern was built between the 16 gels (two replicates of a four gel set for each genotype) and the software detected 312 spots to be matching. 26 spots were found to be differentially expressed with an intensity difference of more than two-fold (Table 2, Figures 3, 4). A significant genotype effect was shown by 7 spots out of which 4 were not affected by treatments (Table 3). A significant treatment effect was shown by 19 spots. Restoration of optimum N conditions resulted in the recovery of some of the down-regulated spots. In addition to this, we found 3 spots that showed treatment as well as genotype effect (Table 4).

**Table 1:** **Variations in protein spot expression in PEHM-2 and HM-4 genotypes of maize**.

**Description**	**No. of spots**
Total number of spots matched	312
Total number of differentially expressed spots	26
Significant genotype effect	7
Significant treatment effect	19
Decrease in intensity under N starvation	10
Increase in intensity under N starvation	9
Spots showing both treatment and genotype effect	3
Down regulated spots fully recovered upon restoration of N in PEHM2	7
Down regulated spots fully recovered upon restoration of N in HM4	0
Up regulated spots recovered to normal upon restoration of N in PEHM2	5
Up regulated spots recovered to normal upon restoration of N in HM4	5

**Table 2:** **Differential expression of leaf proteins in PEHM2 and HM4 geno types of maize under low-N and N restoration conditions**.

**Spot No.**	**SC %**	**Mr/PI**		**Protein name**	**Metabolic pathway**	**Cellular component**	**Differential accumulation in N starvation**		**Status after recovery of N**				**Accession No.**
		**Cal**.	**Exp**.				**PEHM-2**	**HM-4**	**PEHM**		**HM4**		
									**3rd day**	**5th day**	**3rd day**	**5th day**	
A1	71	48079/6.29	51002/6	Ribulose bis phosphate carboxylase/ oxygenase activase, chloroplastic	Energy (photosynthesis)	Chloroplast	Down regulated	Down regulated	+	++	–	+	Q9ZT00
A2	52	103521/5.74	101501/5.2	Pyruvate phosphate dikinase 1, chloroplastic	Photosynthesis/ C4 acid pathway	Plastid/ Chloroplast	Down regulated	Down regulated	+	++	+	+	Isoform 1:P11155-1 Isoform 2: P11155-2
A3	6	47127/4.8	50391/4.3	Phospho enolpyruvate carboxylase	Photosynthesis C4 acid pathway	Cytosol	Up regulated	Up regulated	Recovered to normal	Recovered to normal	Recovered to normal	Recovered to normal	P04711
A4	30	53435/5.88	51000/6.1	Ribulose bis phosphate carboxylase	Calvin cycle, CO_2_ fixation, Photorespiration, Photosynthesis	Chloroplast	Down regulated	Down regulated	+	++	–	+	O03042
A5	44	61604/5.6	62031/5.3	Oxygen evolving enhancer protein 1, chloroplastic	Photosynthesis	Chloroplast membrane/ Thylakoid	Up regulated	Up regulated	Recovered to normal	Recovered to normal	Recovered to normal	Recovered to normal	P12359
A6	36	34706/6.06	32141/6.3	6 phosho gluconate dehydrogenase	Pentose phosphate shunt	Cytosol	Up regulated	Up regulated	Recovered to normal	Recovered to normal	Recovered to normal	Recovered to normal	O60037
A7	19	23123/4.8	20615/5.1	23 kDa polypeptide of PS II	Photosynthesis	Chloroplast (thylakoid membrane)	Up regulated	Up regulated	No change	No change	No change	No change	BAA08564
A8	55	41706/6.1	41215/6	ATP synthase gamma chain, chloroplastic	ATP synthesis	Chloroplast	Down regulated	Down regulated	+	++	+	+	P29790
A9	54	55773/5	54100/5.4	ATP synthase SU alfa, chloroplastic	ATP synthesis	Chloroplast	Down regulated	Down regulated	+	++	+	+	Q6L3A1
B1	79	53957/5.38	51001/6	ATP synthase SU beta	ATP synthase	Chloroplast	Down regulated	Down regulated	+	++	+	+	P19366
B2	64	35995/6.33	42100/5.9	Malate dehydrogenase, Cytoplasmic, 2	TCA cycle	Cytoplasm	Down regulated	Down regulated	–	–	–	–	P57106
B3	6.1	26029/6.2	21105/6.1	Enolase 2	Glycolysis	cytoplasm	Up regulated	Up regulated	No change	No change	No change	No change	P42895
B4	72	119743/5.1	108761/5	Probable sucrose phosphate synthase 1	Sucrose biosynthesis	Cytoplasm	Down regulated	Down regulated	++	++	+	+	O04932
B5	78	29264/7.2	25183/6.9	Imidazole glycerol- phosphate dehydratase 1	Amino acid biosynthesis	Chloroplast	Up regulated	Up regulated	Recovered to normal	Recovered to normal	Recovered to normal	Recovered to normal	Isoform 1 = P34047-1 2 = P34047-2
B6	52	32354/5.1	33412/4.9	Luminal binding protein 2 (fragment)	ATP binding	Endoplasmic reticulum	Up regulated	Up regulated	Recovered to normal	Recovered to normal	Recovered to normal	Recovered to normal	Q03682
B7	62	67161/6.07	69212/7	Methionine synthase	Biosynthesis of methionine	cytoplasm	Down regulated	Down regulated	–	–	–	–	Q9LMO3
B8	66	60622/4.8	61004/5.2	Apurinic endonuclease redox protein	DNA repair	Chloroplast nucleiod	Up regulated	Up regulated	No change	No change	No change	No change	P45951
B9	56	41876/5.23	40801/5.6	Actin 11	Structural constituent	Cytoplasm/ Cytoskeleton	Down regulated	Down regulated	–	+	–	+	P53496
C1	51	17603/4.1	17800/5	Deoxyuridine 5'-triphosphate nucleotide hydrolase	Pyrimidine metabolism	Cytosol	Up regulated	Up regulated	No change	No change	No change	No change	Q9STG6

*+, slight recovery; ++, full recovery; –, no recovery*.

**Table 3:** **Genotypic differences in the expression of protein under controlled conditions**.

**Spot No**.	**SC %**	**Mr/PI**		**Protein name**	**pathway**	**Cellular component**	**Differential protein expression within genotypes**		**Accession No**.
		**Cal**.	**Exp**.						
							**PEHM-2**	**HM-4**	
C5	46	84106/5	80012/5.4	Probable cyclic nucleotide gated ion channel 17	Transport	Cell membrane	More	Less	Q8L7Z0
C6	52	12408/4.7	13088/5	Auxin repressed 12.5 KDa protein	Auxin regulating pathway	Cytosol	Less	More	Q05349
C7	43	42547/6.04	41022/6.2	Sedoheptulose-1,7- bisphosphatase	Carbohydrate biosynthesis, Calvin cycle	Chloroplast	More	Less	P46285
C8	46	45498/7.1	45201/6.8	PSII stability/ assembly factor HCF 136	Photosynthesis	Chloroplast	More	Less	Q525A8

**Table 4:** **Proteins expressed differentially between genotypes and treatments both**.

**Spot No**.	**SC %**	**Mr/PI**		**Protein name**	**Metabolic pathway**	**Cellular component**	**Differential expression in low -N treatment**		**Status after restoration of N supply**		**Accession No**.
		**Cal**.	**Exp**.								
							**PEHM-2**	**HM-4**	**PEHM**		
									**3rd day**	**5th day**	
C2	48	26785/7.1	21663/6.9	Chap-20	Chaperon	Chloroplast	Upregulated	No change	Recovered to normal	Recovered to normal	O65282
C3	46	68656/5.22	69112/5.6	HSP70	Stress response	Chloroplast envelope	Upregulated	No change	Recovered to normal	Recovered to normal	Q9C7X7
C4	28	8253/6	8103/6.3	Subtilisim chymotrypsin inhibitor CI-1C	Protease inhibitor	Cytosol	Upregulated	No change	Recovered to normal	Recovered to normal	P01054
	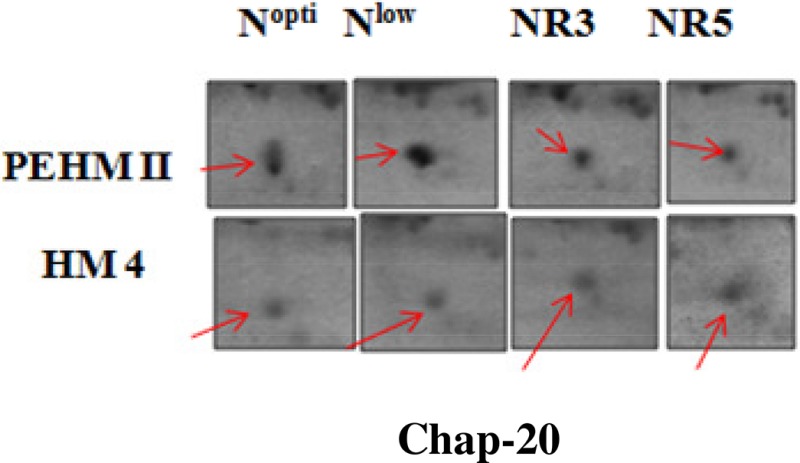				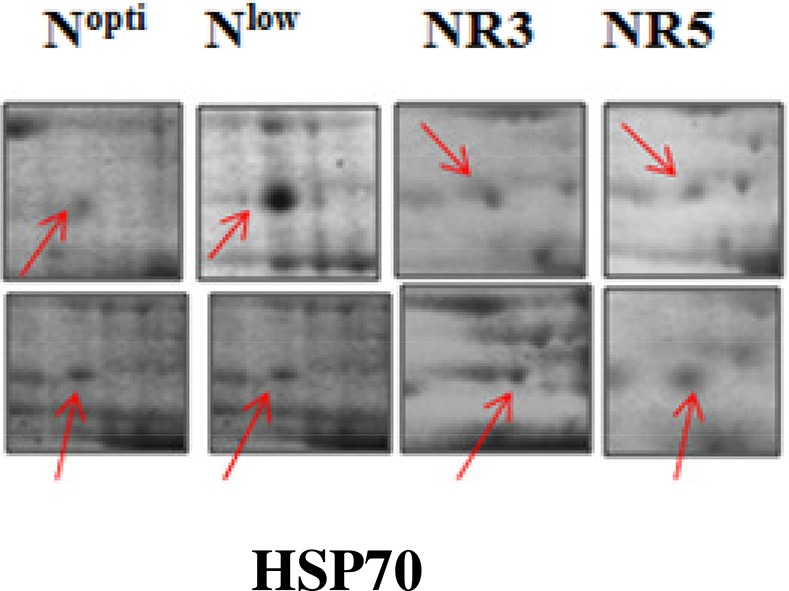				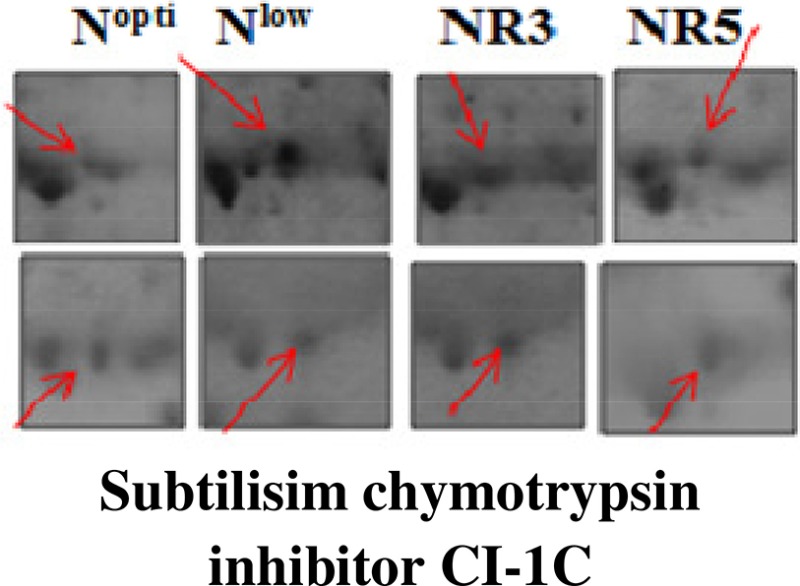		

### Differential expression of proteins in low-N tolerant and low-N sensitive maize genotypes under N starvation and restoration

Twenty-six different protein spots showed significant differences to low N treatment. Three protein spots among them also showed significant genotypic differences and were involved in defense. Nineteen protein spots showed differential expression exclusively for N treatment. Of these, 10 proteins were found to be down-regulated and 9 were up-regulated under low N conditions (Table 2).

Spot A1 has been identified as ribulosebisphosphate carboxylase/oxygenase activase. Intensity of this protein was found to be decreased to one-third under low-N in both the genotypes, compared to control. Upon restoring optimum N concentration to the treated plants, intensity of the protein spot was recovered by one fold on the 3rd day of recovery and got fully restored to normal by the 5th day in PEHM-2. In HM-4 however, intensity of this spot was recovered by only one fold on both 3rd and 5th days of recovery. Pyruvate phosphate dikinase 1 (spot A2) was down regulated by 4-fold under low-N in both the genotypes. Restoration of the optimum concentrations of N to the treated plants resulted in recovery of the protein. In case of T1, partial recovery in the PEHM-2 genotype was observed on the 3rd day after N restoration and the intensity of the protein was restored to that in control on the 5th day. The HM 4 genotype showed partial recovery of the protein spot on both 3rd and 5th days of N restoration. Spot A3 has been identified as phosphoenolpyruvate carboxylase. An up-regulation in its level of expression was observed under low N. The level of increase was 3-fold in case of low N both the genotypes. Upon restoring optimum concentrations of N to the treated seedlings, the levels of PEP carboxylase in both the genotypes were found to be lowered to that of control, immediately after the 3rd day of recovery. On the 5th day also, same intensity of the protein was observed. Spot A4 is ribulose-1,5-bisphosphate carboxylase. There was down-regulation in the protein expression level under low N. Decrease in expression was 3.5 fold in PEHM-2 and 4.0 fold in HM-4. Restoration of the optimum level of N to the treated seedlings resulted in recovery of ribulosebisphosphate carboxylase levels. It was observed that on the 3rd day of recovery the protein intensity increased by one fold in both the genotypes. At the 5th day the protein intensity was found to recover fully in PEHM-2, however, in HM-4 it remained the same as on the 3rd day. Oxygen evolving enhancer protein 1 is found to be spot A5. This protein was up-regulated under low N in both the genotypes. When the optimum concentration of N was restored to the treated seedlings, expression level of the protein was returned to normal immediately after the 3rd day of recovery in both the genotypes. Spot A6 has been identified as 6-phosphogluconate dehydrogenase. Under low N condition, this protein was found to be up-regulated in both the genotypes. Upon restoring the optimum N concentration to the treated seedlings, expression level of the protein returned to normal after the 3rd day of recovery in both the genotypes and was the same for the 5th day post recovery as well. Spot A7 was identified as 23 kDa polypeptide of PS II. Low-N caused an up-regulation of three-folds in this protein in both the genotypes. On the 3rd day and 5th day of N recovery to the treated seedlings, the expression levels were still three times as in the control, which means restoring the optimum concentration of N had no effect on the up regulated expression level of the protein. Spot A8 is ATP synthase gamma chain. A two-fold decrease in the expression level was observed under low N in both PEHM-2 and HM-4. Restoration of the optimum N concentration to the treated seedlings resulted in recovery of the down regulated protein after 5th day in PEHM 2 and HM 4, however, in HM-4 the protein did not recover fully. Spot A9 has been identified as ATP synthase SU alpha. This protein is also a subunit of ATP synthase and is involved in the synthesis of ATPs in the chloroplast. A two-fold decrease in the expression level of both the genotypes was observed under low N. This protein spot showed recovery on 5th day of the restoration of optimum N concentration to the treated seedlings. Genotype PEHM-2 was recovered fully while as the genotype HM-4 showed recovery of the protein intensity partially. Spot B1 has been identified as ATP synthase SU beta. This protein is another subunit of the enzyme ATP synthase involved in the synthesis of ATPs in the chloroplast. This protein subunit was also down-regulated by two-folds under low N in both the genotypes. Restoration of optimum N concentration to the treated seedlings did not show recovery of the protein spot intensity till the 3rd day but was recovered on the 5th day. Genotype PEHM-2 was recovered fully while as the genotype HM 4 showed recovery of the protein intensity partially. Spot B2 is malate dehydrogenase 2. This is a cytoplasmic protein and is a part of the tricarboxylic acid (TCA) cycle. Intensity of the protein spot was decreased by 3-folds under low N in both the genotypes. No effect on the protein spot intensity was shown by the restoration of optimum N concentrations to the treated seedlings. Both the genotypes continued to have a low expression of the protein spot at the 3rd and 5th days of N restoration. Spot B3 has been identified as Enolase 2. Both the genotypes showed a two-fold increase in the intensity of the protein spot under low N. Restoration of optimum N concentration to the treated seedlings had no effect on intensity of the protein spot. Both the genotypes continued to show an increased expression level of the protein spot at the 3rd and 5th days of N restoration. Spot B4 is probable sucrose phosphate synthase 1. There was a 3-fold decrease in the expression level of this protein spot under low N. Upon restoration of the optimum N concentration, the PEHM-2 genotype showed full recovery of the protein spot intensity on the 3rd day and remained same on the 5th day as well. Genotype HM-4 also showed recovery by one-fold but did not reach the control value. Spot B5 is imidazoleglycerol- phosphate dehydratase 1. Low-N treatment resulted in a 3-fold upregulation of the protein spot in both the genotypes. Restoration of optimum N concentrations to the treated seedlings resulted in recovery of the protein spot intensity to that in control. This was the same for both the genotypes. Spot B6 has been identified as luminal binding protein 2 (fragment). This protein aids in ATP binding inside the endoplasmic reticulum. Low-N resulted in a 2-fold upregulation of the protein spot in both the genotypes. Upon restoration of the optimum N concentration to the treated seedlings, intensity of the upregulated protein spot was found to return to its intensity in the control soon on the 3rd day and maintained the same intensity on the 5th day as well. This was true for both the genotypes. Spot B7 is methionine synthase. Low-N treatment resulted in the down-regulation of this enzyme to half of its intensity in control in both the genotypes. Restoration of optimum N concentration showed no effect on the protein spot intensity on either of the 3rd or 5th days of recovery in both the genotypes. Spot B8 is apurinic endonuclease redox protein. This protein is present in the nucleoid of the chloroplast and is involved in DNA repair. Low-N resulted in the 3-fold upregulation of this protein in both the genotypes. Restoration of optimum N concentrations showed no effect on the protein spot intensity on either of the 3rd or 5th days of recovery in both the genotypes. Spot B9 is actin-11. This protein is a part of the cytoskeleton and is located in the cytoplasm, it is a structural constituent of the cell. Low N resulted in the down-regulation of this protein to its half in both the genotypes. Restoration of optimum N concentration showed no effect on the protein spot intensity the 3rd day of recovery but on the 5th day the spot intensity rose by one fold in both the genotypes. Spot C1 is deoxyuridine 5′-triphosphate nucleotidohydrolase. Low N resulted in the down-regulation of this protein to half in both the genotypes. Restoration of optimum N concentration showed no effect on the protein spot intensity on either of the 3rd or 5th days of recovery in case of HM-4, however it showed partial recovery in genotype PEHM-2. Spot C2 is of Chap-20. Low-N treatment caused a 2-fold upregulation of this protein in both the genotypes. The protein spot intensity was recovered as soon as optimum N concentrations were restored such that on the 3rd and 5th days of recovery the protein spot intensities for both the genotypes matched the intensity of control. Spot C3 is of HSP 70. Low N resulted in a 2-fold upregulation of this spot in both the genotypes. The protein spot intensities were recovered immediately upon restoration of optimum N concentrations. On the 3rd and 5th days of recovery the protein spot intensities for both the genotypes were same as that of control.

Spot C4 is probable cyclic nucleotide gated ion channel 17. This protein showed differential expression between the two genotypes only and was not affected by N treatments. This protein was found to be three times more intense in PEHM-2 as compared to HM-4. Spot C6 is of auxin repressed 12.5 kDa protein. This protein showed differential expression between the two genotypes only and was not affected by N or P treatments. The expression level of this protein was three times more in HM-4 control when compared with the control of PEHM-2. Spot C7 is of sedoheptulose-1,7-bisphosphatase. This protein showed differential expression between the two genotypes only and was not affected by N treatments. Expression of this protein is two times more in PEHM-2 than that of HM-4. Spot C8 is PS II stability/assembly factor HCF 136. This protein showed differential expression between the two genotypes only and was not affected by N treatments. PEHM-2 expressed this protein 1.5 times more than that of HM-4.

## Discussion

Thirty-two maize genotypes were extensively screened for low-N tolerance using physiological and biochemical markers and two single cross hybrids were identified. Important attributes contributing to low-N tolerance were identified. Cluster analysis has been considered as a powerful tool for selecting efficient genotype in a multiple-trait crop breeding (Khoshgoftarmanesh et al., 2012). From cluster analysis it was observed that PEHM-2 is low-N tolerant genotype and it belonged to cluster VI. HM-4 has been identified as low-N sensitive genotype and classified in cluster I.

### Functional characterization of the differentially expressed proteins

**Figure 5:**
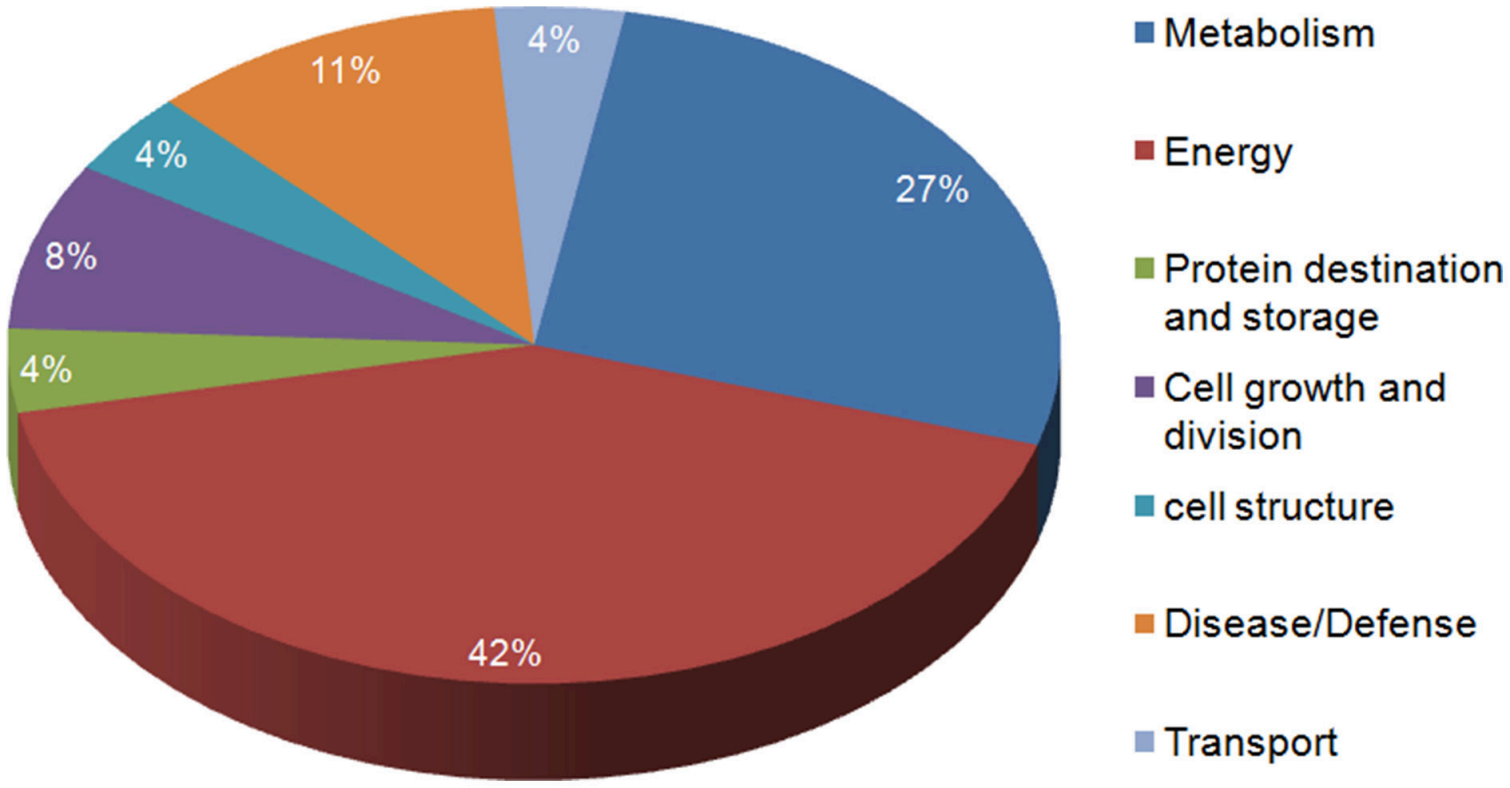
**Functional characterization of the identified proteins**.

In the present study, the highly up-regulated and down-regulated proteins were categorized into seven groups based on their biological functions. A considerably higher number of proteins (10) were down-regulated. Most of these belonged to energy (42%) and metabolism (27%), this was expected as the main functions of leaves (energy harvesting, conversion and storage) are driven by N. However, a smaller number of proteins involved in defense (11%), were observed to be up-regulated more than two-fold. Few proteins belonging to other classes like cell structure, protein destination and storage, cell growth and division and transport also varied in expression under low N conditions (Figure 5).

### Distribution of proteins in sub-cellular compartments

**Figure 6:**
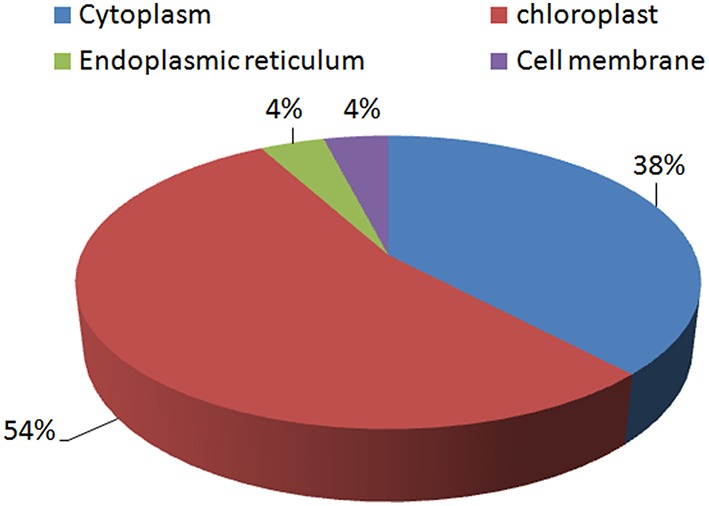
**Distribution of the identified proteins according to their sub-cellular locations**.

Most of the proteins identified were found to be chloroplastic proteins (54%). A significant number of proteins were cytoplasmic proteins as well (38%). Very few proteins were found to be located in the endoplasmic reticulum (4%) and the cell membrane (4%; Figure 6).

### Differential expression of proteins of energy and metabolism of PEHM-2 and HM-4 genotypes under N starvation and its restoration

As expected, most of the proteins that showed differential expression under low N belonged to energy and metabolism. Ribulose-1,5-bisphosphate carboxylase/oxygenase activase (Spot A1) was down-regulated at low N in both PEHM-2 and HM-4. This enzyme plays the double catalytic function of a carboxylase for mediating the carbon dioxide assimilation and an oxygenase for catalyzing the first step of photorespiratory pathway in plants (Kim et al., 2009). Decrease in Rubisco activase under N deprivation was also observed in wheat (Bahrman et al., 2004) and in *Arabidopsis* (Wang et al., 2012). Kim et al. (2009) also found a decrease in the LSU of Rubisco under low N. Down regulation of Rubisco activase suggests that the photosynthetic apparatus undergoes extensive degradation under N deprivation (Wang et al., 2012). Ribulose bisphosphate carboxylase/oxygenase (Spot A4), the rate-limiting enzyme in carbon fixation by photosynthesis (Makino et al., 1985), was also found to be down- regulated under N starvation. Due to low RCA, a decrease in the Rubisco pool was evident since RCA is involved in the activation of rubisco carboxylase. Pyruvate phosphate dikinase (PPDK, Spot A2) is a key enzyme in C4 photosynthetic pathway (Osteras et al., 1997) and is involved in the formation of acetyl CoA. Down-regulation of PPDK under low N was observed in both PEHM-2 and HM-4 genotypes of maize. PPDK was also found to be down-regulated under N deprivation in *P. tricornutum* (Yang et al., 2013a). PEP carboxylase (Spot A3) was found to be upregulated in both the genotypes under low N. Accumulation of PEP carboxylase has also been reported to play a role in adaptation of wheat seedlings to abiotic stress (Ashraf and Harris, 2013). Increase in PEP carboxylase under low N is suggested to optimize the use of available phosphoenol pyruvate carboxylase (PEP carboxylase) for fixation of carbon and thereby keep the pyruvate for acetyl coA formation (Yang et al., 2013b). A subunit of oxygen evolving complex of PSII is the oxygen evolving enhancer protein (Spot A5). It is involved in the stability of PSII, playing a significant role in photosynthesis (Bjorkman et al., 1988). Low-N conditions resulted in the up-regulation of this protein in both HM-4 and PEHM-2 genotypes of maize. It is suggested that on exposure to N stress, oxygen evolving complex proteins are degraded and release oxygen evolving enhancer protein as degradation product to help the plant to adapt to the adverse conditions (Downton et al., 2006). 6-phoshogluconate dehydrogenase (6PGA, Spot A6) stimulates the decarboxylation of 6-phosphogluconate to ribulose-5-phosphate and CO_2_ and a molecule of NADPH is generated. This reaction is a key step to the pentose phosphate pathway (Serres and Nguyen, 1988), an energy saving shunt to the glycolysis. In this study 6PGA was found to upregulate under low N conditions. This might be the plant's strategy to conserve energy and survive better in low N conditions. Spot A7 was found similar to the 23 kDa polypeptide of PS II. It is known to be involved in increasing the binding efficiency of Cl^−^ and Ca^2+^ cofactors required for photosynthetic water splitting activity (Seidler, 1996; Bricker and Burnap, 2005). This polypeptide was found to be up-regulated under low N conditions in both the genotypes. Prinsi and co-workers found down-regulate of this protein when plants growing on low N were supplied with ${\text{NO}}_{3}^{-}$ (Prinsi et al., 2009). A strong up-regulation in this polypeptide in low N treated samples of rice was also observed (Song et al., 2011). Spot A8, A9, and B1 were similar to the gamma chain of ATP synthase, alpha subunit of ATP synthase and beta subunit of ATP synthase, respectively. ATP synthase is an important enzyme of ATP biosynthetic pathway. It is involved in the production of ATP from ADP in presence of a proton gradient across the membrane and takes part in photophosphorylation (Hou et al., 2009) and more importantly in the synthesis of ATP, energy currency of the cell. In our study all the alpha, beta and gamma subunits of ATP synthase were down-regulated under low-N condition in both the genotypes. Differential expression of ATP synthase beta subunit was also observed in wheat (Bahrman et al., 2004). The transcript level of ATP synthase gamma chain was also found to decrease under water stress and heat stress (Andjelkovi and Mici, 2011). Salt stress, however, was found to induce the beta subunit of the same enzyme. Malate dehydrogenase (Spot B2) catalyzes the final step of the citric acid cycle and transforms malate to oxaloacetic acid (OAA) by producing NADH. The expression of this spot was down-regulated in PEHM-2 and HM-4 under low N. This enzyme was moderately increased by the increase in nitrate level in *Arabidopsis* and wheat (Wang et al., 2000; Bahrman et al., 2004). Enolase 2 (Spot B3) is involved in catalyzing the dehydration of 2-phosphoglycerate to form phosphoenolpyruvate. Concentration of enolase was reported to enhance in response to abiotic stresses (Wang et al., 2000). In the present study, low N caused the upregulation of this enzyme, similar results were obtained in tomato roots by Wang et al. (2001). Umeda et al. (1994) isolated an enolase EST in N starved rice cells. Sucrose phosphate synthase 1 (Spot B4) catalyzes the first step of sucrose formation in plants (Lunn et al., 2000). Low N resulted in the down-regulation of this enzyme in PEHM-2 and HM-4. Intensity of this protein has been reported to decrease under drought also (Benešová et al., 2012). Imidazoleglycerol-phosphatedehydratase (Spot B5) belongs to the family hydrolyases and is involved in amino acid biosynthesis catalyzing the seventh step in the biosynthesis of histidine in plants. In the present study, an increased expression of this enzyme was observed under low N condition. Being a part of the histidine biosynthesis pathway, imidazole glycerol phosphate, constitutively with other enzymes of the pathway has shown a role in imparting nickel tolerance to hyper accumulator plants (Ingle, 2011). Luminal binding protein-2 (Spot B6) is a member of the HSP 70 family and is localized to the ER. It is involved in proper protein folding and protein translocation into the ER lumen (Wang et al., 2010). In this study, luminal binding protein 2 was up regulated under low N conditions which might imply the need for accelerated protein folding due to N stress. Since N starvation causes abiotic stress, this protein may act as a chaperon for maintaining the structure of other proteins. Methionine synthase (Spot B7) is involved in the transformation of homocysteine to methionine, which is a precursor for S-adenosyl methionine (SAM; Bahrman et al., 2004). In bacteria and algae, the MetE was reported to be inactivated under oxidative and temperature stress conditions (Hondorp and Matthews, 2004). These results suggest that methionine synthase can be a target of abiotic stress and phytohormones. Spot C1 has been identified as deoxyuridine 5′-triphosphate nucleotidohydrolase, which is a precursor of thymidine nucleotides and reduces the intracellular concentration of dUTP, thereby preventing uracil for incorporation into DNA. Under low N, this enzyme showed down-regulation in both the genotypes. Abdelmohsen et al. (2014) have correlated the stress related down-regulation of deoxyuridine 5-triphosphatenucleotidohydrolase gene with DNA damage. Spot 26 was found corresponding to probable fructose bis-phosphate-aldolase 2. This enzyme catalyzes the conversion of D-fructose 1,6-bisphosphate to glycerone phosphate and D-glyceraldehyde 3-phosphate in glycolysis. Under low N, it did not show any significant change. Rocco et al. (2013) found this enzyme to be upregulated under cold treatment and downregulated under heat stress. As the content of sedoheptulose-bis-phosphatase in thylakoid membranes increases at low N conditions, spot A24 might have a role in conferring improved photosynthesis and growth to it at low N availability. Significant increase in this protein was also reported in *Vitis vinifera* under drought stress (Cramer et al., 2013). Chloroplastic PSII stability/assembly factor HCF 136, is responsible for stabilizing and activating the PSII complex (Meurer et al., 1998; Plucken et al., 2002). Its abundance in PEHM-2 may therefore imply activated PSII and hence efficient photosynthesis as compared to HM-4.

Most of the differentially expressed proteins related to energy and metabolism were recovered after the seedlings were restored with optimum N concentrations. However, some proteins took slightly longer to recover fully than the rest. Also, few proteins did not show recovery at all. The enzymes of photosynthesis that were down regulated namely rubiscoactivase (A1), pyruvate phosphate dikinase 1 (A2) and ribulosebisphosphate carboxylase (A4) responded to N restoration, and were found to be recovered completely. It is suggested that under N deprivation the photosynthetic apparatus undergoes extensive degradation, its reassembly upon return of normal N conditions might take some time, that is why on the third day of recovery slight improvement in Rubisco activase, pyruvate phosphate dikinase and Rubp carboxylase is observed until they get fully recovered on the 5th day. HM-4 being the less tolerant genotype, it took time to recover its photosynthetic machinery fully. Among the upregulated proteins, photosynthesis related spots, spot A5 (Oxygen evolving enhancer protein 1) resumed to its control value immediately upon restoration of N supply. This may be attributed to the fact that under N stress, the oxygen evolving complex degrades into oxygen evolving enhancer proteins to help the plant deal with low N, removal of N stress, therefore, causes the oxygen evolving enhancer proteins to return to normal. Spot A6 (6 phoshogluconate dehydrogenase), that was seen to upregulate under N stress as a strategy for the plant to survive better under low N conditions, was seen to resume to its control value upon the return of optimum N. Similarly, the enzymes involved in metabolism ATP synthase gamma, alpha and beta subunits (A8, A9 and B1), sucrose phosphate synthase (B4) also recovered upon return of the optimum N. Proteins like Imidazole glycerophosphatedehydratase 1 (B5) and Luminal binding protein 2 were also recovered to normal immediately after N restoration, further validating the given differential expression to be due to low N.

### Differential expression of proteins involved in cell growth and division of PEHM-2 and HM-4 genotypes under N starvation and its restoration

Spot B8 was found corresponding to the apurinic endonuclease redox protein. The amino-terminal domain of this protein is highly charged and is involved in DNA repair because it increases the affinity of the protein for DNA (Babiychuk et al., 1994). An up- regulation of this protein under low N was found in both the genotypes and the intensity of the spot was not resumed to the control value even upon the restoration of optimum N concentrations. Auxin repressed 12.5 kDa protein (Spot C6), a part of the auxin signaling pathway, showed differential expression between the genotypes and was found to be more intense in PEHM-2. Tian et al. (2008) have reported lower auxin contents in the phloem sap and roots of maize supplied with high N. The lower intensity of auxin repressed 12.5 kDa protein may, therefore, correlate with increased root growth and thus efficient N uptake in PEHM-2.

### Differential expression of cell structure proteins of PEHM-2 and HM-4 genotypes under N starvation and its restoration

Actin-11 (Spot B9) is a globular protein which is the monomeric subunit of microfilaments and one of the major components of the cytoskeleton (Katam et al., 2013). Low N resulted in a decrease of actin-11 expression in both the genotypes and its intensity was seen to improve back upon restoration of optimum N. Water stress has also been found to cause down-regulation of the actin gene (Katam et al., 2013).

### Differential expression of protein destination and storage (folding and stability) of PEHM-2 and HM-4 genotypes under N starvation and its restoration

Chaperonin 20 (Chap-20) protein (Spot C2) was found to increase under low N in both the genotypes and was resumed to the control value upon optimum restoration of N in our study. Chap-20 belongs to chaperonins, a subclass of molecular chaperons that assist in correct folding of proteins in the cell normally (Ellis and Hartl, 1996) and under stress (Rao and Lund, 2010). It has been found that the overexpression of the groESL operon enabled *Anabaena* to combat salt stress better, particularly protecting the photosynthetic pigments (Chaurasia and Apte, 2009). Intensity of a 59.7 kDa chaperonin protein increased under N starvation in *Oscillatoria willei* (Saha et al., 2003).

### Differential expression of disease/defense responsive proteins in PEHM-2 and HM-4 genotypes under N starvation and its restoration

Nitrogen stress caused upregulation of HSP 70 (Spot C3). Restoration of N supply resumed the protein to its control state. The overexpression of HSPs in response to stress is an important adaptive strategy in providing tolerance to plants (Gazanchian et al., 2007; Lee et al., 2007; Zhang et al., 2010). Yang et al. (2013a) reported an upregulation in HSP 70 in *Phaeodactylum tricornutum* under N deprivation. Lin HSP 16.45 has been found to have a role in plant response to multiple abiotic stresses (Mu et al., 2013). The overexpression of MTS HSPs and CaHSP 26 genes in tobacco exhibited tolerance to increased temperature and chilling stress, respectively. An upregulation of HSP 70 was also reported in *Phaeodactylum tricornutum* under N deprivation (Yang et al., 2013a).

### Differential expression of transport protein in PEHM-2 and HM-4 genotypes under N starvation and its restoration

Spot C5, probable cyclic nucleotide gated ion channel 17, is a member of the non-selective cation channel (NSCC) transport proteins. These are Ca^2+^-permeable cation transport channels, involving in the uptake of essential and toxic cations, calcium ion signaling, pathogen defense, and thermo-tolerance in plants (Nawaz et al., 2014). In *Physcomitrella patens*, the cyclic nucleotide gated calcium channel (CNGC) CNGCb gene encodes a component of cyclic nucleotide gated Ca^2+^ channels that is responsible for its acquired thermotolerance (Finka et al., 2012). In addition to this, Tunc-Ozdemir et al. (2010) have reported cyclic Nucleotide-Gated Channel 16 to be critical for stress tolerance in pollen reproductive system. A change in the expression of such a protein has been shown to alter the Na^+^, K^+^, Ca^2+^ ions flux into the tissues (Gobert et al., 2006; Guo et al., 2008). Increased Na^+^,K^+^, Ca^2+^ may be needed by a plant for maintaining the cationic balance under low-N concentration. In our study, the cyclic Nucleotide-Gated Channel 17 protein was found to have a greater intensity in the PEHM-2 genotype, which may be of benefit to the plant for being able to tolerate N stress better.

### Differential expression of protein by genotypic effect

Seven protein spots showed significant differences in the expression pattern among the two genotypes under control condition. Out of these 7 protein spots, 4 spots were not affected by treatments, thus showing genotype effect specifically (Table 3). One of the spots was more intense in HM4 and 3 were more intense in PEHM2. MALDI-TOF identification and the GO annotations of the identified spots revealed that these proteins were probable cyclic nucleotide gated ion channel 17 (spot C5), auxin repressed 12.5 kDa protein (spot C6), sedoheptulose-1,7- bisphosphatase (spot C7), PSII stability/ assembly factor HCF 136 (spot C8). The expression of all these proteins was found to be more in PEHM-2 than HM-4, except auxin repressed 12.5 kDa protein, which was found to be more intense in HM-4. Remaining three of the seven protein spots showed varied accumulation under different N levels. These were identified as Chap-20 (spot C2), HSP70 spot C3) and subtilisin chymotrypsin inhibitor (spot C4). Increased abundance of these spots was noted for PEHM-2 genotype and low N conditions as well. Spot C5 identified as cyclic nucleotide gated ion channel 17 is a member of the non-selective cation channel (NSCC) transport proteins. A change in the expression of such a protein has been shown to alter the Na^+^, K^+^, Ca^2+^ ions flux into the tissues (Hampton et al., 2005; Ali et al., 2007). Increased Na^+^,K^+^, Ca^2+^ may be needed for maintaining the cationic balance under low N concentration. The lower expression of auxin repressed 12.5 kDa protein in low-n tolerant genotype may be correlated with increased root growth and thus efficient N uptake in PEHM-2. As the content of sedoheptulose bisphosphatase in thylakoid membranes increases at low N condition, this protein might have a role in conferring improved photosynthesis and growth to PHEM-2 at low N availability. Chloroplastic PSII stability/assembly factor HCF 136 is responsible for stabilizing and activating the PSII complex (Meurer et al., 1998; Plucken et al., 2002). Its abundance in PEHM2 may, therefore, imply activated PSII and, hence, efficient photosynthesis as compared to HM-4. These proteins might have a role in distinguishing the mechanism of N uptake among the two genotypes.

## Conclusion

Nitrogen (N) has been a critical input for plant nutrition, hence crop productivity. In a bid to increase crop production, farmers apply a lot of N in various forms of the N-fertilizers. However, the applied N is only partially utilized by plants. The unused N causes severe environmental pollution and huge financial loss. This is because commonly cultivated varieties of crop plants are usually high N responsive. Development of crop varieties with high nutrient use efficiency is imperative for sustainable agriculture. Understanding how plant genes respond to low N stress is essential for formulating approaches to manipulating genes for improving nutrient use efficiency. Efforts made in this study through proteomic analysis of low-N tolerant and low-N sensitive maize genotypes may provide such information about the candidate gene(s) for stimulation or development of varieties which can be able to grow and yield well even at low level of N. This study clearly demonstrated that the N limitation caused differential expression of protein in low-N tolerant and low-N sensitive maize genotypes. Leaf proteome analysis reported 25 polypeptides were affected by N deficiency. Four polypeptides showed differential expression as a genotypic effect irrespective of N treatment. Most of the proteins that were differentially accumulated in response to N treatments were involved in photosynthesis and metabolism, affirming the relationship between N and carbon metabolism. In addition to this, greater intensity of some defense proteins in the low N tolerant genotype was also found to have a possible role in helping it tide over low N conditions. Finding out these variable proteins and their apparent role in conferring N use efficiency is the first step toward a more effortful task. These low N-responsive proteins should contribute to a better understanding of adaptation to low N stress in maize and hence pave the way to develop the plants that can grow and yield well at low N supply.

## Author contributions

MN, RP, AA, MQ conceived and designed the experiments. MN and AA performed the experiments. MN, KV, RP, MI, TS, GA, AA analyzed the data. MN, RP, MQ, MI, TS, AA, GA, and KV wrote the paper.

### Conflict of interest statement

The authors declare that the research was conducted in the absence of any commercial or financial relationships that could be construed as a potential conflict of interest.

## Abbreviations

*Z. mays*: *Zea mays*
2-DE: Two-dimensional gel electrophoresis
Rubisco: ribulose-1,5-bisphosphate carboxylase/oxygenase
NUE: nitrogen use efficiency, N, nitrogen
NR3: 3rd day N restoration
NR5: 5th day N restoration.
