# Supplementary material for: Nitrogen-Deficiency Stress Induces Protein Expression Differentially in Low-N Tolerant and Low-N Sensitive Maize Genotypes
Source: Front Plant Sci. 2016 Mar 21;7:298. doi: 10.3389/fpls.2016.00298 (PMC4800187; doi:10.3389/fpls.2016.00298)
Supplement: Supplementary file 2 [file Table2.DOCX]

Supplementary Table 2.Analysis of variance (ANOVA) on physiological and biochemical traits of maize genotypes grown under nitrogen stress. N – nitrogen levels, G – genotype, CD – critical difference at 1%

| Traits | *P* value | | | CD at 0.01 | | |
| --- | --- | --- | --- | --- | --- | --- |
|  | N | G | N X G | N | G | N X G |
| Shoot length | 0.000*** | 0.000*** | 0.000*** | 1.42 | 5.69 | 8.05 |
| Root length | 0.000*** | 0.000*** | 0.000*** | 2.08 | 8.32 | 11.76 |
| Shoot dry weight | 0.000*** | 0.000*** | 0.000*** | 0.047 | 0.188 | 0.266 |
| Root dry weight | 0.001** | 0.000*** | 0.000*** | 0.012 | 0.051 | 0.072 |
| Total biomass | 0.000*** | 0.000*** | 0.000*** | 0.055 | 0.218 | 0.309 |
| Leaf area | 0.000*** | 0.000*** | 0.000*** | 9.58 | 38.3 | 54.17 |
| Root-to-shoot ratio | 0.000*** | 0.000*** | 0.000*** | 0.04 | 0.16 | 0.227 |
| Chl a | 0.000*** | 0.000*** | 0.000*** | 0.049 | 0.195 | 0.276 |
| Chl b | 0.000*** | 0.000*** | 0.000*** | 0.059 | 0.235 | 0.332 |
| NR | 0.000*** | 0.000*** | 0.000*** | 0.087 | 0.348 | 0.492 |
| GS | 0.000*** | 0.000*** | 0.000*** | 0.082 | 0.327 | 0.462 |
| N concentration | 0.000*** | 0.000*** | 0.000*** | 0.054 | 0.214 | 0.302 |
| Total N uptake | 0.000*** | 0.000*** | 0.000*** | 1.574 | 6.295 | 8.903 |
| NUE | 0.000*** | 0.000*** | 0.000*** | 1.041 | 4.165 | 5.891 |
| Photosynthesis | 0.000*** | 0.000*** | 0.000*** | 1.51 | 6.042 | 8.545 |
