# Supplementary material for: Nitrogen-Deficiency Stress Induces Protein Expression Differentially in Low-N Tolerant and Low-N Sensitive Maize Genotypes
Source: Front Plant Sci. 2016 Mar 21;7:298. doi: 10.3389/fpls.2016.00298 (PMC4800187; doi:10.3389/fpls.2016.00298)
Supplement: Supplementary file 3 [file Presentation1.PPTX]

## Slide 1
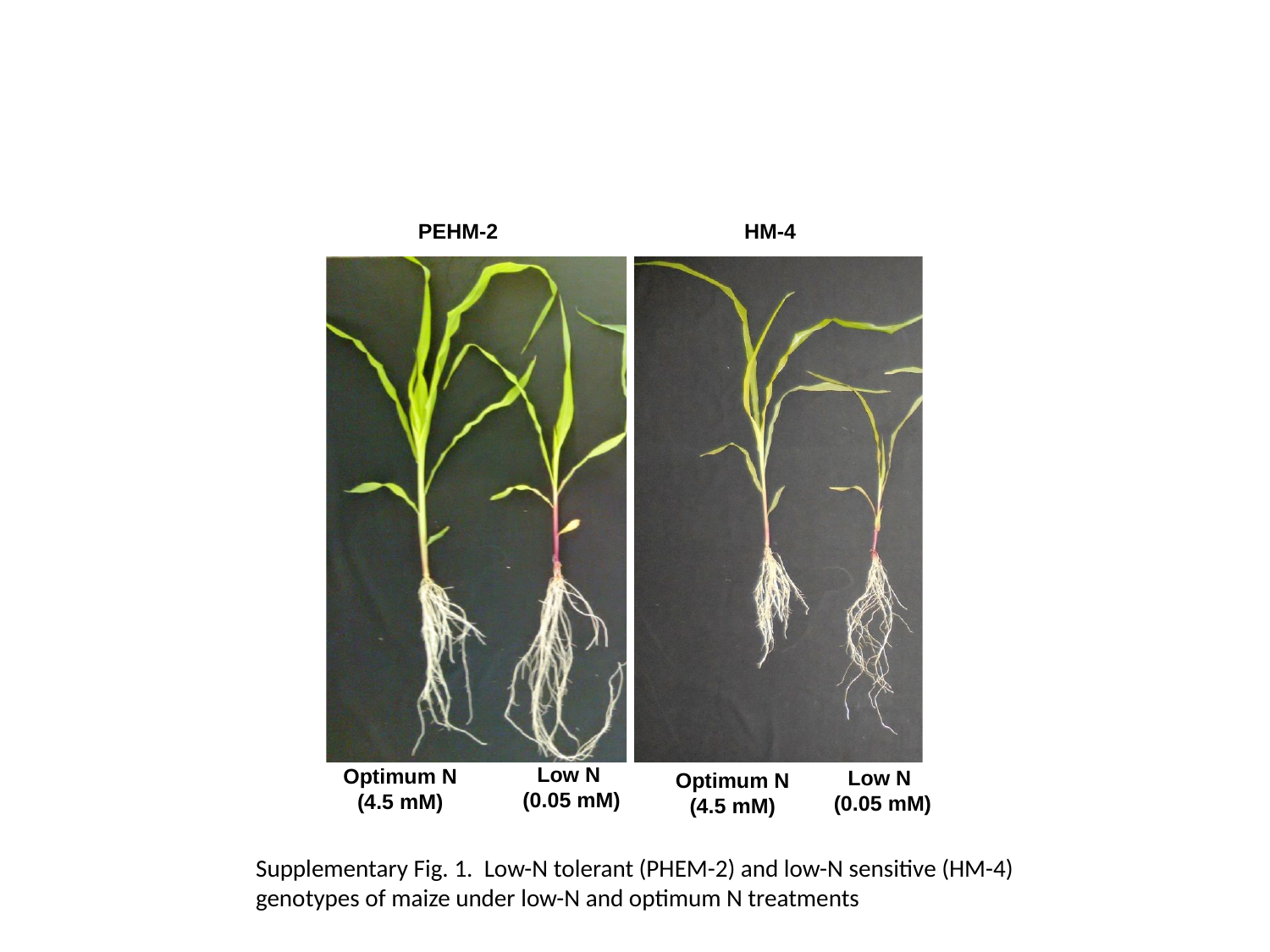

HM-4
PEHM-2
Low N
(0.05 mM)
Optimum N (4.5 mM)
Low N
(0.05 mM)
Optimum N (4.5 mM)
Supplementary Fig. 1. Low-N tolerant (PHEM-2) and low-N sensitive (HM-4) genotypes of maize under low-N and optimum N treatments
