# Supplementary figures and images for: Nitrogen-Deficiency Stress Induces Protein Expression Differentially in Low-N Tolerant and Low-N Sensitive Maize Genotypes
Source: Front Plant Sci. 2016 Mar 21;7:298. doi: 10.3389/fpls.2016.00298 (PMC4800187; doi:10.3389/fpls.2016.00298)

## Slide 1
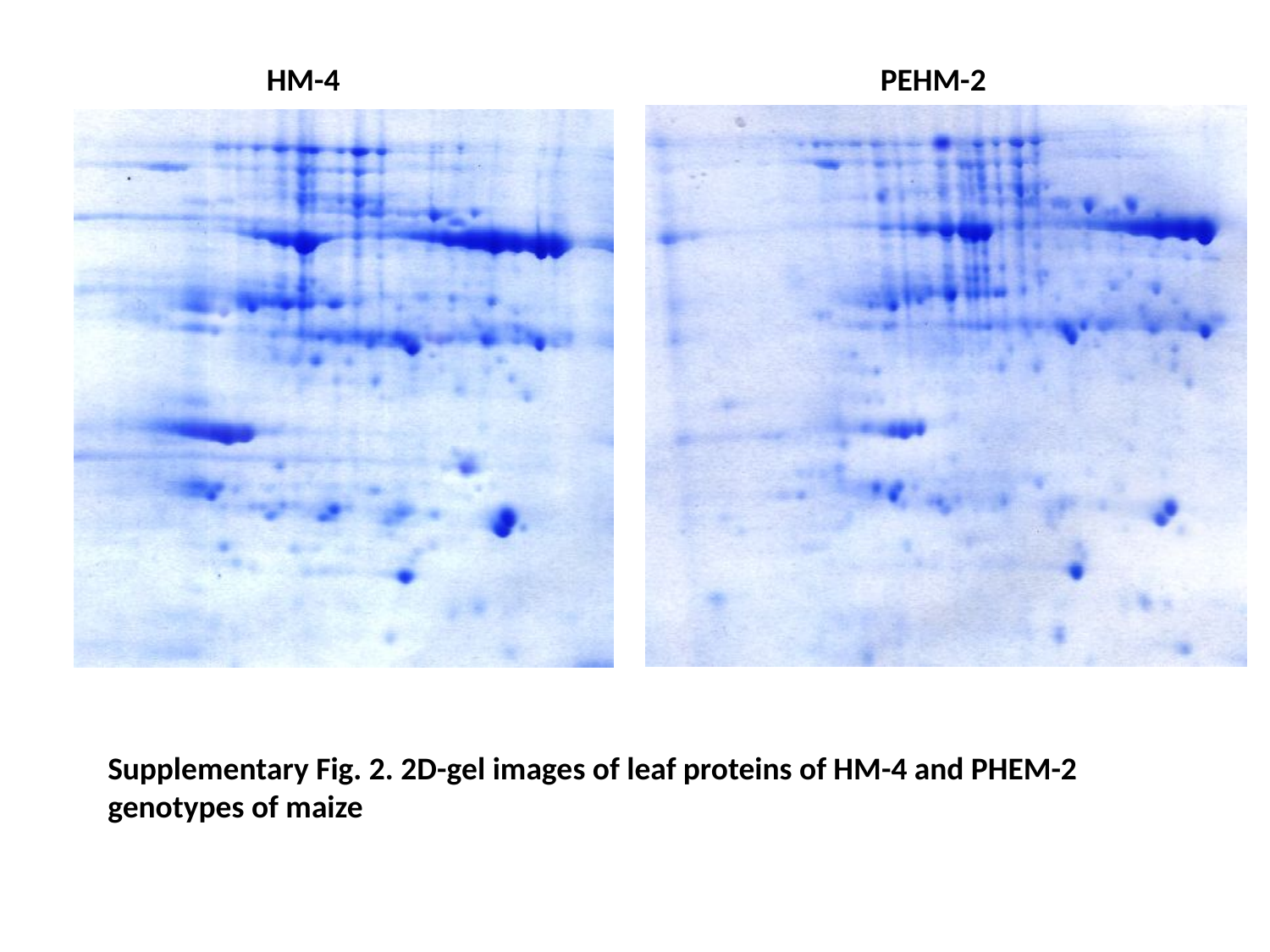

HM-4
PEHM-2
Supplementary Fig. 2. 2D-gel images of leaf proteins of HM-4 and PHEM-2 genotypes of maize

Supplement: Supplementary file 4 [file Presentation2.PPTX]
